# Synchrotron X-ray diffraction investigation of the surface condition of artefacts from King Henry VIII’s warship the *Mary Rose*

**DOI:** 10.1107/S1600577520001812

**Published:** 2020-04-15

**Authors:** Mark G. Dowsett, Pieter-Jan Sabbe, Jorge Alves Anjos, Eleanor J. Schofield, David Walker, Pam Thomas, Steven York, Simon Brown, Didier Wermeille, Mieke Adriaens

**Affiliations:** a Department of Physics, University of Warwick, Coventry CV4 7AL, UK; b Department of Chemistry, Ghent University, Krijgslaan 281-S12, B-9000 Ghent, Belgium; c The Mary Rose Trust, College Road, HM Naval Base, Portsmouth PO1 3LX, UK; d Department of Physics, University of Liverpool, Liverpool L69 7ZX, UK; e XMaS – The UK CRG, ESRF – The European Synchrotron, CS40220, 38043 Grenoble Cedex 9, France

**Keywords:** cultural heritage, SR-XRD, corrosion, brass, conservation, marine archaeology

## Abstract

Synchrotron X-ray diffraction (XRD) measured on the XMaS beamline at the ESRF was used to characterize the alloy composition and crystalline surface corrosion of three copper alloy Tudor artefacts recovered from the undersea wreck of King Henry VIII’s warship the *Mary Rose*. The XRD method adopted has a dynamic range ∼1:10^5^ and allows reflections <0.002% of the height of major reflections in the pattern to be discerned above the background without smoothing. Laboratory XRD, scanning electron microscopy–energy dispersive spectroscopy, synchrotron X-ray fluorescence and X-ray excited optical luminescence–X-ray near-edge absorption structure were used as supporting techniques, and the combination revealed structural and compositional features of importance to both archaeology and conservation. The artefacts were brass links believed to be fragments of chainmail and were excavated from the seabed during 1981 and 1982. Their condition reflects very different treatment just after recovery, viz. complete cleaning and conservation, chemical corrosion inhibition and chloride removal only, and distilled water soaking only (to remove the chlorides). The brass composition has been determined for all three at least in the top 7 µm or so as Cu(73%)Zn(27%) from the lattice constant. Measurement of the peak widths showed significant differences in the crystallite size and microstrain between the three samples. All of the links are found to be almost chloride-free with the main corrosion products being spertiniite, sphalerite, zincite, covellite and chalcocite. The balance of corrosion products between the links reflects the conservation treatment applied to one and points to different corrosion environments for the other two.

## Abstract 2

A new method using a high-sensitivity X-ray camera for a large-range diffraction pattern is presented; it confirms the effectiveness of over 35 years of conservation treatment for brass artefacts from the Mary Rose.

## 1. Introduction   

On 19 July 1545, Tudor warship the *Mary Rose* sank in the Solent close to the entrance to Portsmouth Harbour in the UK during a battle with the French (Marsden, 2019 ▸). Around 500 lives were lost and attempts to recover the ship failed at the time (Marsden, 2019 ▸; Hildred, 1988 ▸). A significant fraction of the hull sank deep into the silts covering that area of the bed of the Solent and was, with its contents, preserved as a remarkable record of Tudor naval engineering and shipboard life (Rule, 1982 ▸; Jones, 2003 ▸; Marsden, 2003 ▸). In 1982 the remaining part of the hull was recovered, and it is now housed in the Mary Rose Museum in Portsmouth, UK, alongside some of the >19 000 artefacts that were also recovered from the seabed (Marsden, 2003 ▸; Dobbs, 1995 ▸; Mealing, 2013 ▸). Artefacts of wood (including the hull), metal and many other materials were remarkably well preserved by the anaerobic conditions in the Eocene clays into which she sank. The wood and its conservation in particular have been studied in detail (Schofield *et al.*, 2016 ▸).

**Figure 1:**
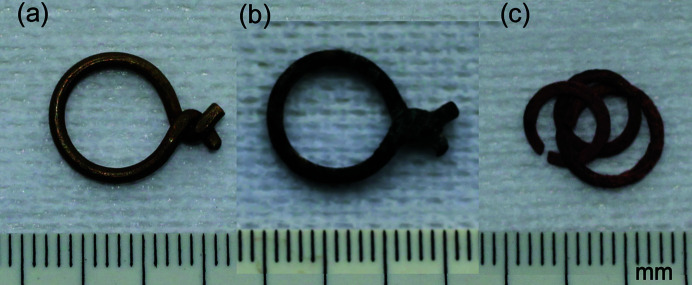
Brass samples believed to be various forms of chainmail links. (*a*) MR81A1436, the cleaned and conserved link; (*b*) MR81A2249, similar but not cleaned; and (*c*) MR82A6000, having the appearance of copper.

As part of a continuing scientific investigation of the recovered artefacts, here we report on the use of synchrotron X-ray diffraction (SR-XRD) supported by laboratory XRD, scanning electron microscopy–energy dispersive spectroscopy (SEM-EDS) and some synchrotron X-ray fluorescence (XRF) to study the surface condition of brass artefacts in various states of conservation 33–34 years after their recovery (measurements carried out 07/2015 and 03/2016). The objective was to make detailed but non-destructive measurements of the surface chemistry of three small items identified as links from chainmail (Fig. 1 ▸). Two of these were twisted loops of brass wire [MR81A1436 and MR81A2249, Figs. 1 ▸(*a*) and 1(*b*)] and one appeared to be copper or a copper alloy in the form of three linked flat washers [MR82A6000, Fig. 1 ▸(*c*)]. The first two were of drawn wire, whereas the last was probably produced by stamping from a sheet. Many individual and linked examples of these artefacts were recovered, some in undersea exploration of the wreck and some when she was raised (Jones, 2003 ▸). MR81A1436 had been subjected to cleaning and conservation treatment in 1981, the nearly identical MR81A2249 had been soaked in aqueous benzotriazole (BTA) solution to inhibit further corrosion, and MR82A6000 had no treatment apart from soaking in distilled water in 1989 (see Table 1 ▸). Since treatment, all three have been stored in an environmentally controlled facility where the relative humidity was kept below 30% at a temperature of approximately 25°C. Therefore, a key goal was comparison of the state of corrosion on the surfaces, especially between fully and partially conserved items with the former likely to be showing corrosion formed over the last 38 years and the latter evolved from the original corrosion on the seabed.

In the techniques used here, the elemental composition revealed by SEM-EDS and XRF was used to aid in the interpretation of the diffraction patterns. However, the combination only gives information on the crystalline materials present. Therefore, in a parallel study we used a microscope (XEOM 1) (Dowsett *et al.*, 2015 ▸) based on X-ray excited optical luminescence (XEOL) to examine the copper *K*-edge X-ray near-edge absorption structure (XANES). Some of these data have been published elsewhere (Dowsett *et al.*, 2015 ▸; Sabbe *et al.*, 2014 ▸).

The principal reason for using SR-XRD is that the artefact surfaces are strongly curved and rough and the artefacts are also quite small. The centimetre-sized beam in a typical laboratory diffractometer will result in peak broadening and splitting because of integrated height errors and the statistics will be poor as the majority of the beam will miss the artefact. Significant background will result from scattering from backing surfaces even if these are selected so as not to contribute sharp peaks to the pattern. On the other hand, we have shown that the high power densities in synchrotron microbeams cause rapid damage to the types of compound expected on copper alloys (Adriaens *et al.*, 2013 ▸). The XMaS beamline (Brown *et al.*, 2001 ▸) delivers its flux into a beam of millimetre dimensions as projected into the on-sample footprint and the power density had not caused problems in many previously reported measurements on copper corrosion products. Nevertheless, the laboratory XRD acquired first served as a check on the stability of the surface chemistry during synchrotron measurements.

## 2. Experimental   

### 2.1. Sample details   

The three samples were a subset from twelve artefacts all subjected to the same analytical process (materials: brass, lead, silver, bronze and pewter). Here, we focus on this subset because they are similar materials, all in the form of links but with different conservation treatments and surface composition. Nevertheless, the SR-XRD from other samples was examined along with the data discussed in detail here to establish sources of background signal. Details of the three samples together with their initial treatment are shown in Table 1 ▸. The concretion (*i.e.* calcium carbonate and silicates from microfauna) which often completely encapsulates marine artefacts, was minimal because copper is toxic to marine organisms (Jones, 2003 ▸).

The samples were removed from storage in the *Mary Rose* reserve collection and individually packed in acid-free paper envelopes. The set was placed in an hermetically sealed box with desiccant and remained therein for transport to the ESRF and storage during analysis. Only one sample at a time was unpacked to minimize the risk of cross-contamination. Samples were handled with disposable nitrile gloves and occasionally with soft plastic tweezers.

### 2.2. Analytical techniques   

The artefacts were initially examined using XRD on a Panalytical X’Pert Pro MPD at The University of Warwick, UK. The instrument was equipped with a curved Johansson monochromator providing pure focused Cu *K*α_1_ radiation and a solid-state PIXcel linear detector. A variable slit was used to keep the footprint size constant at 10 mm × 15 mm in the 2θ scan. The samples were placed in a shallow well in an off-cut silicon wafer and held in place with 6 µm-thick Ultralene film. Sample rotation at 60 rpm was used for MR82A6000 because the interlinking of the trio of washers gave rise to some height-related peak splitting. The main results came from 17 h scans across the range 10–100° 2θ.

SR-XRD was carried out at the UK CRG beamline XMaS at the ESRF (Brown *et al.*, 2001 ▸). The samples were mounted in a custom-made precision vice with prismatic jaws and surface pegs made of a soft engineering plastic: acetal co­polymer [Fig. S1(*a*) in the supporting information]. Acetal copolymer has an easily recognizable diffraction pattern with sharp reflections [Fig. S1(*b*)] and care was taken where possible not to hit the mounting surfaces during analysis. A reference material in the form of a 12 mm-diameter × 2 mm-thick 99.96% pure copper disc (Goodfellow Metals Ltd, UK) was added to the set of samples. This disc was polished with 1 µm alumina powder on a lint-free pad mixed into a paste in 2-propanol and ultrasonically cleaned in 2-propanol.

The 8.5 keV beam was incident at a fixed angle of 5.5° to enhance the surface specificity and focused to a footprint approximately 1.7 mm wide × 1.8 mm long on the sample surface. The detector was a Pilatus3 R 300K camera (Dectris, Switzerland) mounted on the 2θ arm of the Huber goniometer (Huber Diffraktiontechnik GmbH) on XMaS. The sensor of the camera was positioned 1011.6 mm from the centre of the beam footprint (verified by the displacement of the Cu 111 reflection as the camera angle was stepped in 2θ), and its face was centred laterally on the beam and mounted tangential to the arc of the 2θ scan as shown in Fig. S2. The sensor is made from three modules, 487 pixels wide and 195 pixels high, separated by two intermodule gaps each 17 pixels wide to make a total array of 487 × 609 pixels. The pixels are 172 µm squares and the central pixel therefore subtends 0.00974° in 2θ which determines the basic resolution of the system. The long side was aligned with the direction of scan and the camera was stepped in 1.042° increments across the 2θ range of 5° to 90° to cover the total range of 2° to 93° (taking account of the finite extent of the camera face). The scan step deliberately places the intermodule gaps in one image over mid-module pixel rows in the previous or following image. Since the gaps would cause significant glitches in recovered 1D patterns unless filled, we copy strips from the mid-module rows of image *n* and paste them into the intermodule gaps of image *n* − 1. The pasted strips are copied from an identical 2θ range in image *n* to that subtended by the gaps in image *n* − 1. Because the camera face is flat there is, in principle, a difference in the range of 2θ subtended by whole pixels in different parts of the face. However, the resulting error in our copy–paste was ∼2% of the pixel height and was ignored. (Note however, the flatness of the camera face is fully accounted for in the image-merging process described below.) Figs. S3(*a*) and S3(*b*) show open and filled gaps in the raw Pilatus image. A fast shutter was mounted in the beam path so that the samples were only irradiated during imaging.

Each complete scan consisted of 82 images accumulated for 100 s per image in the case of the *Mary Rose* samples and 150 s for the reference. Processing of the set was done automatically using the custom software package *esaProject* (Dowsett *et al.*, 2012 ▸). Each image was first normalized to the signal from an ion chamber mounted after all beam-defining slits [Fig. S1(*a*)] and gap-filled. It was then mapped into a merged image in γ–2θ space (where γ is the out-of-plane scattering angle) with a ‘pixel’ pitch of 0.01° (user-selectable and being in this case close to the mean angle subtended by all camera pixels across the 2θ range). Since pixels from the camera will not overlap exactly with pixels in the angular map, their precise position in the merged image is calculated and then the intensity is partitioned into the four nearest-neighbour target pixels according to the degree of overlap, assuming the pixels are square. This strategy eliminates the aliasing which would result from rounding to the nearest integer position. The software keeps track of the source pixel fractions added to the destination pixels and sums these. This is essential whether working with individual images or the merged image because the geometry of an image (from a rectangular sensor) after mapping into γ–2θ space leaves the high-2θ end of the image cusped and the low-2θ end convex. The number of pixels contributing to a constant 2θ row therefore varies with 2θ from 1 or 2 to 487 at the ends of a single image and by around 16% at image boundaries in the merged image. The final 1D pattern is therefore integrated along constant 2θ rows in the merged image and normalized to the sum along the integration path. The 1D pattern can also be multiplied by a constant to conserve its original integrated intensity if desired. The entire process can be done without the explicit generation of the merged image, but we retain it because it is essential to be able to inspect the morphology of the diffraction rings (*e.g.* diffuse, spotty, streaky) when interpreting the 1D data. The extraction of the merged image and the 1D pattern takes <7.5 s per image set on a Windows 10 laptop with a 6 core 2.2 GHz CPU. Further processing such as background removal, comparison with reference patterns, measuring peak position and full width at half-maximum (FWHM), and output plotting of the data were carried out using *esaProject*.

XEOL-XANES data were also acquired on XMaS using the broadband photomultiplier (H8259-01, Hamamatsu Photonics KK) fitted to our XEOL full-field microscope system XEOM 1 (Dowsett *et al.*, 2015 ▸; Sabbe *et al.*, 2014 ▸). X-rays were incident at 60° to the surface, and take-off was at 45°. The range 8.905–9.075 keV was scanned using 1 eV steps at 200 s per step (XEOL imaging data not shown here were collected in parallel, hence the large integration time). In addition, XRF measurements were made on the corroded and conserved links MR81A2249 and MR81A1436 in the same beam time allocation. In that case the spectra were measured using a 14 keV beam and a Vortex silicon drift detector (Hitachi High-Technologies Science, America Inc.). The detector was used for XANES at the Cu *K* and Pb *L*_III_ edges and was consequently heavily attenuated by a 0.4 mm-thick aluminium foil in front of the window. Therefore, no elements below iron are detectable and the heavy elements are significantly over-represented in the raw data. The spectrum was therefore corrected for energy-dependent transmission using a curve derived from absorption data on the Centre for X-ray Optics (CXRO) website (Henke *et al.*, 1993 ▸).

The WWW-MINCRYST database (Chichagov *et al.*, 2001 ▸), Inorganic Crystal Structure Database (ICSD), Cambridge Structural Database (CSD) and Crystallography Open Database (COD) were used to obtain reference patterns to aid the identification of compounds observed using XRD. The latter three were searched through *CrystalWorks* via the Daresbury Laboratory UK server. Where necessary, CIF files were converted to theoretical patterns to be read by *esaProject* using *Mercury* (version 4.1.3; Macrae *et al.*, 2008 ▸). Fig. S4 shows the stick patterns for the species identified on the sample surfaces.

SEM-EDS was carried out at the University of Warwick using an EDAX Genesis system on a JEOL6100 SEM. The 20 keV electron beam was scanned over an area approximately 1.5 mm × 1.5 mm. It was only possible to extract semi-quantitative data from the spectra because the samples were rough and inhomogeneous. The detector has an extremely thin polymer window so atomic numbers down to 6 are observable.

## 3. Results and discussion   

### 3.1. Overview and copper reference   

In the case of the SR-XRD, three factors are combined to produce raw data with a dynamic range ∼1:10^5^. These are (i) the combination of the low intrinsic noise and high dynamic range of the sensor, (ii) the integration of ∼3800 pixels into each of the 9500 points on the 1D pattern due to the overlap produced by the scan step, and (iii) the high beam flux. These give rise to patterns where the count in the face-centred cubic (f.c.c.) 111 peaks for copper and α-brass comfortably exceeds 10^8^. At the same time the background level averages ∼10^6^ counts. The statistical fluctuation of the background is ∼0.1% (Poisson statistics), so exceptionally small peaks <0.002% of the f.c.c. reflections are discernible above the background. The 1D patterns are shown as counts versus wavenumber *Q* (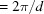) to provide beam energy independent plots. None of the data shown here have been smoothed.

**Figure 2:**
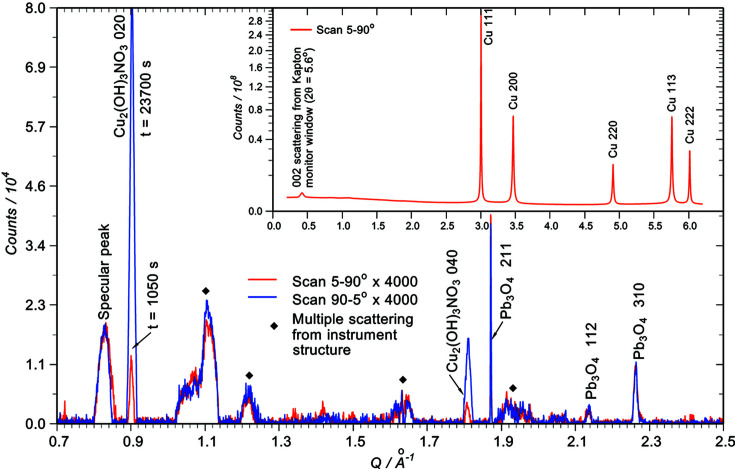
Data from the copper reference (no smoothing has been applied). The inset has been normalized to the beam monitor, then multiplied by a constant to restore the integrated count. The main figure has been background subtracted and shows two consecutive scans, 5–90° then 90–5°. Note the growth of gerhardite at *Q* = 0.9 Å^−1^ and 1.81 Å^−1^ as the scans proceed.

Fig. 2 ▸ shows data for the copper reference. This was scanned twice – first from 5° to 90° and then without pause from 90° back to 5° – to see if there were X-ray induced changes in the surface. The total X-ray dose received by the sample was three times that for any of the artefacts. Fig. 2 ▸ (inset) shows the first scan with a square-root intensity scale to bring up the background. The peaks are quasi-Lorentzian (or Cauchy) in shape, typical of all high-intensity reflections in our data. Apart from normalization to the beam monitor, the data are as collected (with the total count restored after normalization). The main plot (linear scale) shows the region 0.7 < *Q* < 2.5 Å^−1^, from which the background has been subtracted. No other features were visible above the background apart from the main f.c.c. peaks and a peak appearing at *Q* = 0.42 (2θ = 5.6°, see inset). This peak (a diffuse ring in the images, see Fig. S3) is due to 002 forward scattering (Isoda *et al.*, 1981 ▸) at 5.3° from the Kapton exit window on the beam monitor about 60 mm up-beam of the sample. Together with a peak at *Q* = 0.827 Å^−1^ (2θ = 11°) attributed to specular reflection it was common to all the data collected irrespective of the material. The specular peak arose from a diffuse broad line across the camera face. Other features (marked by filled diamonds) were observed only on copper and its alloys. They arose from changes in image intensity with no symmetry (*i.e.* not intersections of the camera face with Scherrer cones). Again they are not indicative of sample chemistry and are most likely due to multiple scattering (*e.g.* of the f.c.c. 111 reflection) off the instrumental structure (such as the goniometer). This is a phenomenon which is almost inevitable because a sensor of this type can receive X-rays from any direction. The labelled chemically related reflections all arose from the integration of diffuse or spotty rings in the images. With one exception, no copper compounds were detected on the reference surface, indicating that any native oxide was too thin to detect by these means. However, reflections corresponding to a small patch of minium (lead tetroxide, Pb_3_O_4_) were identified. The copper disc is produced using the same tooling as our lead reference discs so there is potential for some cross-contamination observable in high-sensitivity data.

The one copper compound observed was gerhardite [Cu_2_(OH)_3_NO_3_], where the 020 and 040 reflections were present. Notably they increase over time. The 020 peak on the second scan taken after ∼23 700 s of X-ray exposure is almost eight times larger than in the first scan after ∼1050 s. Clearly there has been an X-ray induced surface reaction arising from natural humidity and the generation of NO_*x*_ and O_3_ by the beam. However, no such effect was seen on the *Mary Rose* samples which were protected by the pre-existing corrosion and the lower dose.

### 3.2. Atomic composition data   

Table 2 ▸ shows the approximate atomic composition in wt% of the top few micrometres of the link surfaces from SEM-EDS and XRF spectra. The sample columns run from most to least contaminated (in terms of atomic composition other than Zn and Cu) running from left to right. The corrected and background-subtracted XRF data are shown in Fig. S5. The arsenic, selenium and lead data in the table come from the XRF. SEM-EDS spectra were measured in three places on MR81A2249 and MR81A1436; on the twisted part, and on two places around the loop. There was no obvious correlation with position except that sodium on MR81A2249 was correlated with chlorine with the highest level at one place on the loop. This suggests a speck or patch of halite (NaCl) was in the SEM field of view, which is unsurprising considering that the artefacts were recovered from and stored in close proximity to the sea. Two measurements were made on MR82A6000 links. The table shows the average of the measurements where ‘Trace’ implies <0.1% and the ± values show the extremes.

The arsenic, selenium and lead concentrations on the conserved link are less than one-tenth of those on the corroded surface. Arsenic and selenium are commonly found in Eocene silts (Garnit *et al.*, 2017 ▸) and clays and, moreover, selenium and lead are known to have been transported into the Solent by tributary rivers such as the Test and the Itchen (Measures & Burton, 1978 ▸). The difference between MR81A2249 and MR81A1436 suggests that the heavy elements are mostly absorbed into the corrosion and the original concretion from the silt, the sea water or both. However, in the case of lead, XRD data show that the metal is present on the surface as small inclusions in both cases.

Comparing MR81A2249 and MR81A1436, the oxygen, sulfur and chlorine levels are all significantly higher on the former as one would expect from the lack of treatment. On this link, the high carbon and nitro­gen levels probably come from BTA adsorbed into the corrosion layer. However, for MR81A1436 the low nitro­gen concentration suggests a thin residue of BTA on the metal surface whereas the high level of carbon is likely to be from the silicone oil. The silicon level on this link is 3–7 times higher than on the others consistent with this coating.

The copper and zinc concentrations (wt%) on MR81A2249 and MR81A1436 links varied considerably over the three spots analysed with the former at Cu (8–26%), Zn (5–22%) and the latter at Cu (40–49%), Zn (14–17%). This is indicative of an inhomogeneous surface combined with the rather surface-specific 20 keV SEM-EDS measurement which will sample principally the top 1–2 µm (Goldstein *et al.*, 1992 ▸).

MR82A6000 clearly contains a significant amount of zinc and its surface composition was more consistent in that regard with Cu (66–67%) and Zn (10–14%). Remarkably, these links are also free from the elements seen on the other two, apart from the 8% oxygen and 4% nitro­gen. Apparently, they had been exposed to a different corrosion environment from the others. Assuming that they were originally recovered with nantokite (CuCl) on the surface, the immersion in distilled water would have decomposed the nantokite, leaving cuprite behind which explains the lack of chlorine and the high oxygen level (Grayburn *et al.*, 2015 ▸; MacLeod, 1981 ▸).

### 3.3. Synchrotron X-ray diffraction: composition and structure of the Tudor brass   

**Figure 3:**
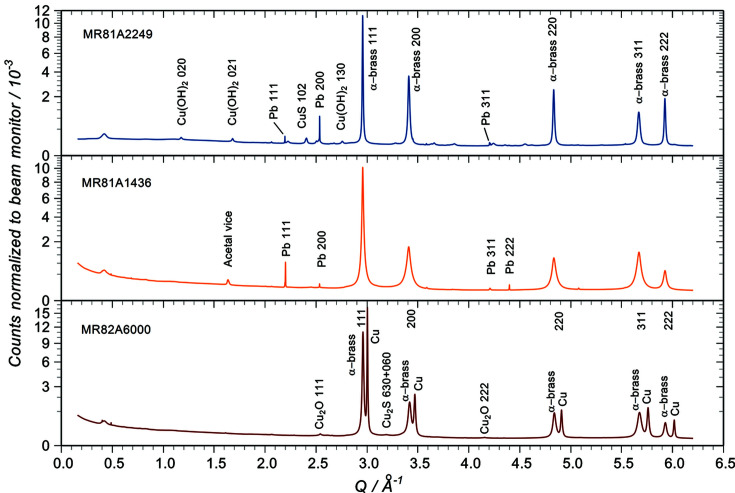
Main diffraction peaks from the three samples showing the brass structure to be face-centred cubic (α-brass). The quasi-Lorentzian shape and symmetry of the peaks is evident. The FWHM values for the reflections from MR81A1436 are visibly larger. The presence of copper metal as well as brass on MR82A6000 is immediately apparent.

Fig. 3 ▸ shows the most intense (*i.e.* the f.c.c.) reflections from the three samples. The peaks are symmetrical with long tails typical of a Lorentzian distribution. The raw intensities are comparable with those for copper, but these data are normalized to the beam monitor without restoration of the total count so that they may be directly compared. The attenuation length of 8.5 keV X-rays in these matrices is ∼26 µm (Henke *et al.*, 1993 ▸), so given the small angle of incidence the sampling depth lies in the range 5–7 µm, notably more than SEM-EDS.

Clearly all the links are α-brass (Voncken & Verkroost, 1997 ▸) and careful examination of the patterns shows that no other brass phase is detectable. However, the double f.c.c. peaks on MR82A6000 are most noticeable and show the presence of both brass and a pure copper phase. Two of the individual links from MR82A6000 were analysed with identical results. Given the appearance of the links, the copper most likely lies over the surface of the brass in a rough layer with a thickness sufficient to contribute strong reflections whilst being reasonably transparent to the beam. By comparing the (monitor-normalized and background-subtracted) intensity of the copper reflections with those from the reference and assuming that the difference is due to the average finite thickness of copper *t* for the link compared with an infinite thickness for the reference, an estimate of *t* can be made using
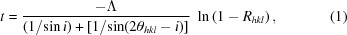
where Λ is the attenuation length (26 µm at 8.5 keV), *i* is the incident angle, 2*θ_hkl_* is the scattering angle for the *hkl* reflection and *R_hkl_* is the ratio of the peak areas from the link and the reference (see supporting information). Averaged across all five reflections and two measurements in different positions, this comes to 1.3 ± 0.3 µm. This layer is probably the result of dezincification of the brass in sea water. However, if the zinc had shown a concentration gradient towards the surface in the top ∼7 µm, the brass reflections would be asymmetric with longer tails on the high-*Q* side. On the contrary, they are symmetric, indicating a sharp interface with the surface copper. This suggests that the α-brass dissolved away and the copper replated onto the surface during the attack by seawater (Scott, 2002 ▸). A similar effect, the appearance of a pure copper surface layer during dezincification of brass in 2% NH_4_Cl, was observed using electron diffraction by Hashimoto *et al.* (1963 ▸). MR81A2249 shows no such copper layer, but the brass reflections are again symmetrical and therefore do not indicate a graded zinc composition towards the surface. Clearly the corrosion environments of these two samples were quite different – possibly MR81A2249 in silt and MR82A6000 exposed to sea water.

From the position of the brass f.c.c. reflections we can determine the average zinc composition at least in the near-surface region. Rendle (1981 ▸) measured the relationship between the brass composition (wt%) for the α-phase and the lattice constant *a* and hence the peak position. His data are replotted in Fig. S7 where a more recent data point from Voncken & Verkroost (1997 ▸) has been added. The parabolic fit shown was used for interpolation to obtain the results in Table 3 ▸, where the lattice constants were determined from the two highest-order reflections measured (311 and 222). The wt% figure quoted here is calculated as zinc + copper = 100% unlike that in the SEM-EDS table which was the fraction of the total including corrosion. In these data, individual peak positions can be determined to less than ±0.005°, which results in an error in the lattice constant of ∼2 × 10^−5^ nm for the relevant reflections, but the actual variation in *a* is clearly larger than this. Other factors such as lattice distortion may determine the peak positions in addition to the zinc content. Taking the largest difference in our *a* values (0.00025 nm from the corroded link), we estimate an error in the zinc concentration as 1 wt% which is in line with Rendle’s error. The links therefore all have a similar alloy composition with 27% zinc.

**Figure 4:**
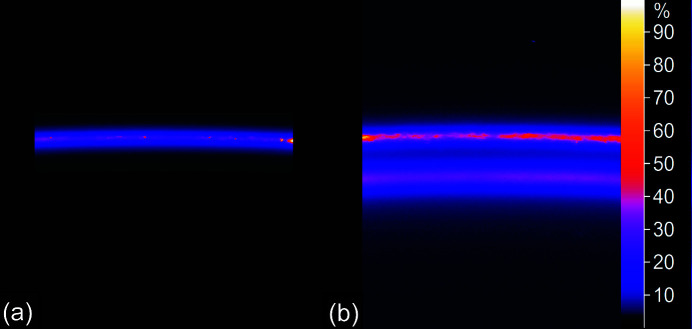
Diffraction rings of the 200 reflections for (*a*) the copper reference and (*b*) MR82A6000 showing both copper and brass. In both cases the copper rings are textured with spots whereas the brass ring is smooth and featureless (ironbow scale).

The SR-XRD images show a distinct morphological difference between the crystallinity of the brass and that of the copper. Figs. 4 ▸(*a*) and 4(*b*) show the 200 reflections from the copper reference and MR82A6000, respectively. In both cases, the copper ring is spotty and streaky indicating the presence of grains of several hundred micrometres in size with preferred orientations (Lipson & Steeple, 1970 ▸) embedded in finer textured material. Conversely, the brass reflection [the lower ring in Fig. 4 ▸(*b*)] is smooth and featureless. This was the case for all α-phase reflections from all three links. The brass grains are therefore random in their orientation and there are no large crystallites to produce texture in the diffraction pattern.

However, there are significant differences in crystal structure between the brass samples revealed by the peak widths; this is evident from Fig. 3 ▸, where those on MR81A1436 have up to three times the FWHM of the copper reference. This can be indicative of a mixture of lattice microstrain, a high level of defects and a small crystal size (Lipson & Steeple, 1970 ▸; Warren, 1990 ▸; Suryanarayana & Norton, 1998 ▸). Moreover, the 111 and 222 reflections are significantly narrower than those for the 200, 220 and 311 planes. Consequently, a plot of *w*cosθ versus sinθ to obtain estimates (Suryanarayana & Norton, 1998 ▸) of the strain η and the apparent crystallite size *s* contains too much scatter to be useful. This is true for all of these samples, even the copper reference. It is likely that the broadening effects can be attributed to cold working of the materials and the additional air abrasion of MR81A1436 producing lattice distortion and defects rather than very small crystal size (Lipson & Steeple, 1970 ▸). At present we do not have enough data to separate the effects of the three probable contributions to line broadening (*e.g.* Ungár, 2004 ▸), so a more detailed analysis is not possible.

### 3.4. Surface chemistry   

**Figure 5:**
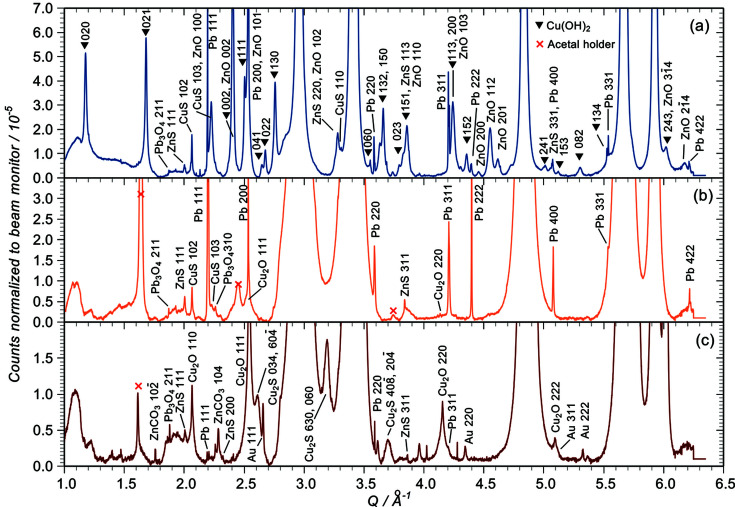
Low-intensity regions of Fig. 3 ▸ after background subtraction. (*a*) MR81A2249. The spertiniite reflections are indicated by filled triangles. (*b*) MR81A1436. The red crosses here and in pane (*c*) show the reflections from acetal copolymer. (*c*) MR82A6000 link, which appeared to be copper but was actually brass.

Fig. 5 ▸ shows the background-subtracted SR-XRD patterns from the three samples at high gain: (*a*) MR81A2249 × 170, (*b*) MR81A1436 × 300 and (*c*) MR82A6000 × 600. A red cross (×) indicates reflections coming from the acetal vice.

#### 3.4.1. Metallic impurities   

Even at low gain (Fig. 3 ▸) the very sharp reflections from lead are evident on the corroded and conserved links. At high gain, reflections out to 422 are visible. Lead is also present at the limit of detection on MR82A6000 [Fig. 5 ▸(*c*)] where some gold is also found. Neither Pb nor Au were detected in by SEM-EDS for this sample, demonstrating the high sensitivity achieved under our SR-XRD measurement conditions.

**Figure 6:**
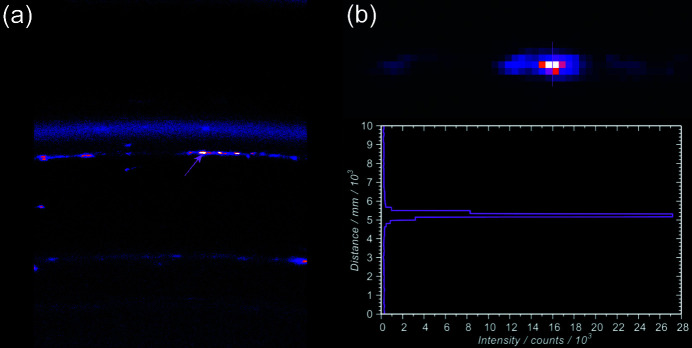
(*a*) Spotty lead ring in the diffraction image taken at 2θ = 29.136° from the corroded link MR81A2249. The ring lies just below CuS 103/ZnO 100 and above CuS 102. (*b*) Detail of the arrowed spot and a section through it showing an FWHM of 1 pixel or less.

The data show that these metals are in the form of small, isolated deposits with few polycrystals and with preferred orientations: the related features in the 2D images are spots which are smaller in extent than the beam footprint. Sometimes these are superimposed on smooth rings, due to, for example, cuprite or zincite (ZnO). Because the deposits are smaller than the aperture formed by the beam they are self-aperturing and their diffraction patterns contain size and distribution information (Lipson & Steeple, 1970 ▸). Firstly, if there were several deposits within the footprint, their scattering would combine to form split peaks rather than the sharp features observed so their density is ∼1 lead particle per 2 mm^2^ from the footprint area. Secondly, the size of the spot in the 2θ direction gives a clue as to its physical size on the sample. Fig. 6 ▸(*a*) shows the spots giving rise to the Pb 111 reflection on the corroded link on the shoulder of the zincite 100 and covellite (CuS) 103 rings. Fig. 6 ▸(*b*) is a detail of the arrowed bright spot in Fig. 6 ▸(*a*) with an intensity map along the section shown as an inset. This is <1 pixel FWHM or <172 µm which transforms to a region <425 µm across on the sample (*i.e.* less than one-fifth of the beam footprint). If the lead had been added deliberately to the brass to improve its ductility we would expect it to be more evenly distributed at the grain boundaries (Bagherian *et al.*, 2016 ▸) and just detectable with SEM-EDS. Moreover, the factor of ten drop in lead content for MR81A1436 with its abrasively cleaned surface compared with MR81A2249, observed using XRF (Fig. S4), suggests that it is on the surface rather than incorporated into the bulk. A similar conclusion applies to MR82A6000 where gold was observed: gold is soluble in both zinc and copper so a metallic gold pattern is unlikely to originate from the bulk.

#### 3.4.2. Corrosion   

The crystalline chemistry of the three surfaces is quite different as far as the balance of corrosion products present is concerned, which is expected in the case of the fully cleaned and conserved link MR81A1436, but which shows that the corrosion environment of MR82A6000 was different from that of MR81A2249. It is also worth remembering that very simple remedial treatment on recovery such as soaking in water can effectively remove chlorides and convert nantokite (CuCl) to cuprite (Cu_2_O) (Grayburn *et al.*, 2015 ▸; Macleod, 1981 ▸), especially on small artefacts where there is no great thickness of metal to absorb seawater.

**Figure 7:**
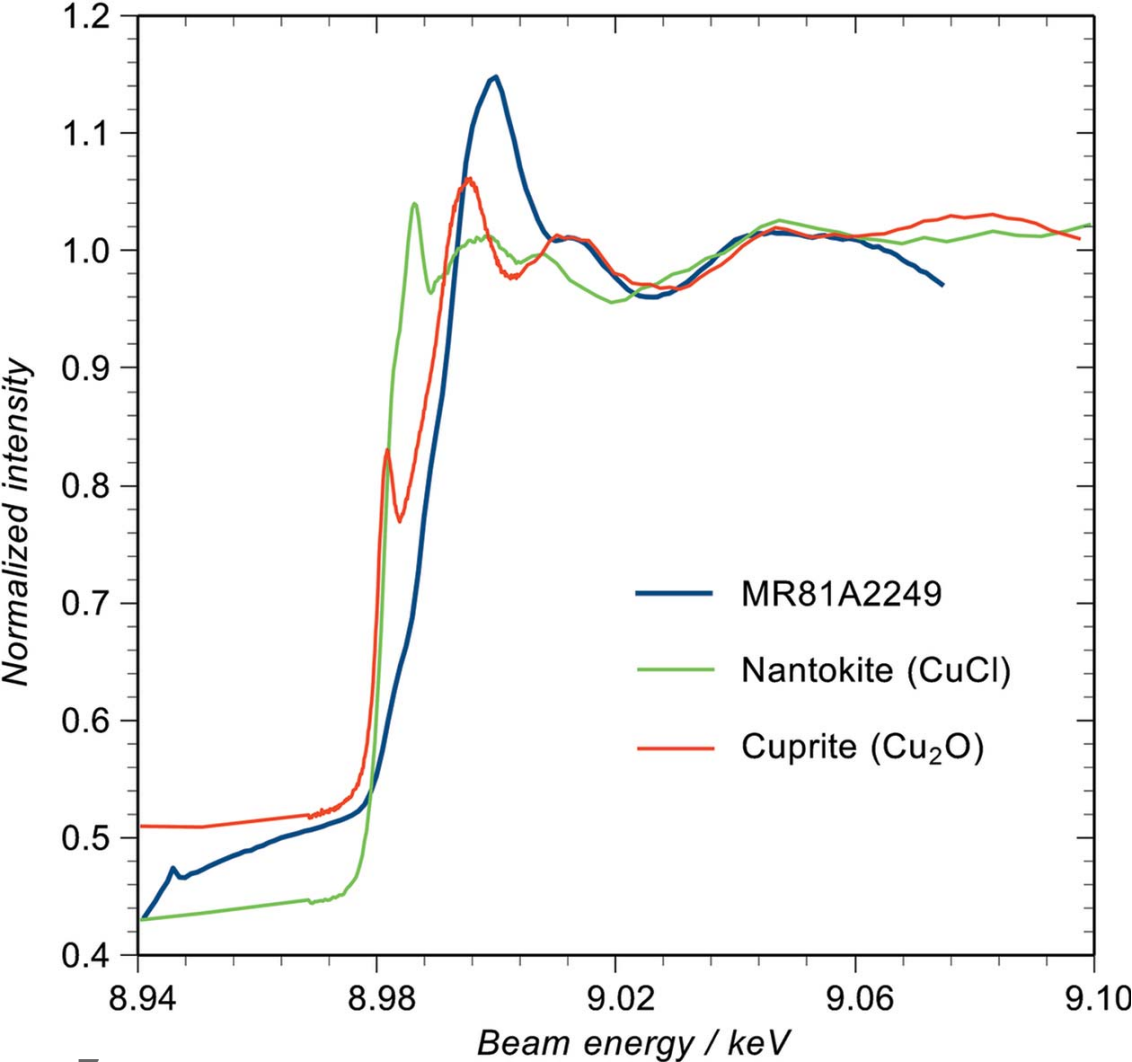
XEOL-XANES spectra from the corroded link (blue curve) with nantokite (green curve) and cuprite (red curve) references. The XEOL-XANES spectra show a strong similarity to the spertiniite XANES spectra published by Farges *et al.* (2007 ▸). There is no sign of nantokite and the edge shift is consistent with a Cu (II) compound such as spertiniite.

Starting with MR81A2249 [Fig. 5 ▸(*a*)], the most intense reflections come from spertiniite [Cu(OH)_2_] (indicated by filled triangles) and zincite with some covellite and a smaller amount of sphalerite (ZnS). The diffraction pattern of sphalerite is very difficult to distinguish from that of nantokite so we cannot be certain from these data that nantokite is not present. However, the nantokite 222 and 420 reflections which do not coincide with sphalerite are invisible (not a definitive argument because they are small and might be below the detection limit). More significantly, there is no sign of paratacamite [Cu_2_Cl(OH)_3_] which forms in air due to the slow hydrolysation of nantokite (Scott, 2002 ▸) and under X-ray bombardment of nantokite in air (Adriaens *et al.*, 2013 ▸). XEOL-XANES data from this sample are shown in Fig. 7 ▸ in comparison with nantokite and cuprite reference spectra taken from powders. There is no sign of the characteristic ‘white line’ (arrowed) of nantokite or any of the other post-edge features, although there is some resemblance to the cuprite reference. Moreover, the edge shift is characteristic of a copper (II) compound. Farges *et al.* (2007 ▸) have published conventional XANES data for spertiniite and our XEOL analysis shows good agreement with their data. Given this, and the absence of paratacamite in the XRD, we conclude that the XRD pattern predominantly contains sphalerite rather than nantokite and the XANES data are characteristic of spertiniite. According to Scott, spertiniite is an unusual corrosion product often amorphous and unstable (Scott, 2002 ▸). Nevertheless, the diffraction shows 18 clear spertiniite reflections, and although several thousand other copper and zinc compounds were tested for a fit to these peaks, it was the only possible candidate. The spertiniite 021 reflection is just visible in the laboratory XRD data (Fig. S6) and so it is not caused by the acetal vice unlike the close reflections in the other two samples. Spertiniite presumably accounts for the blue patches on the link with the sulfides giving rise to the black regions. At the limit of detection is the minium (Pb_3_O_4_) 211 reflection presumably associated with the lead.

Unlike XRD, the XEOL-XANES data show no indication of the brass substrate. This is because the escape depth of the optical photons is only a few hundred nanometres, making the technique much more surface specific (Dowsett *et al.*, 2008 ▸).

Fig. 5 ▸(*b*) shows the pattern from MR81A1436. The treatment with BTA and silicone oil (which are not directly detected in the pattern) has kept the surface free of any significant corrosion since 1982. Once more there is some doubt from these data about the presence or otherwise of nantokite because of its indistinguishability from sphalerite, but again there is no paratacamite hence no evidence of hydrolysis of nantokite. In addition, we show elsewhere (Dowsett *et al.*, 2015 ▸) that Cu *K*-edge XEOL-XANES from the surface of this link does not contain a nantokite signature but is characteristic of cuprite. We therefore conclude that the main residual corrosion products are sphalerite and covellite with some cuprite. The sulfides on both the conserved and corroded links are typical of corrosion in marine sediments in the presence of sulfate-reducing bacteria (Scott, 2002 ▸; Mitchell *et al.*, 2008 ▸).

The data for MR82A6000 are shown in Fig. 5 ▸(*c*). This time the surface corrosion is mainly cuprite and chalcocite (Cu_2_S) with smaller amounts of sphalerite and smithsonite (ZnCO_3_). Again there is no paratacamite, and no chlorine was seen in SEM-EDS so, although we have no XANES from this sample, it is reasonable to choose sphalerite over nantokite here. The predominance of copper corrosion products in this sample is consistent with dezincification to an original depth of several micrometres or even tens of micrometres followed by overgrowth of copper corrosion products on a replated copper-rich surface.

## 4. Conclusions   

A method of using a high-sensitivity X-ray camera to obtain a diffraction pattern over an arbitrarily large range of 2θ has been tested on a set of three artefacts recovered from the wreck of the *Mary Rose* and believed to be chainmail links. The integration of several thousand pixels into a single datum of width 0.01° in 2θ combined with the relatively intense synchrotron beam produces diffraction maxima containing in excess of 10^8^ counts against a background 10^5^–10^6^ counts. Because of the small relative level of Poisson fluctuation in the latter, peaks <0.002% of the main reflections can be discerned. The total acquisition time was <2.5 h per image set. In many cases, acceptable data would be obtained 10 or 100 times faster using this method (excluding settling time for the scan) with a corresponding decrease in X-ray dose.

With the exception of the copper reference sample which received three times the dose of the others, the patinas appeared to be stable under the X-ray dose. The main corrosion products observed were just discernible in patterns obtained with a laboratory diffractometer before the synchrotron measurements. Nevertheless, the growth of gerhardite under the beam on the reference indicates a potential problem and samples should be protected by blanketing in helium and cutting the dose in any future work.

The fully cleaned and conserved link MR81A1436 showed no evidence of significant re-corrosion since recovery and treatment. The other two samples also appeared to have been stable with no detectable chlorine on the linked set of three MR82A6000, and no active chlorine-related corrosion on MR81A2249. No nantokite or copper hy­droxy­chlorides such as paratacamite were detected on any of the samples. On MR81A2249 the chlorine detected in SEM-EDS is associated with sodium so it is likely that this link has a few particles of halite (NaCl) on the surface, although XRD did not detect any halite.

Within the penetration range of the X-rays used (∼5–7 µm), the samples were shown to be brass with a zinc content of 27 wt% as estimated from the lattice spacing. MR82A6000, which we believed from its appearance to be copper, was shown to be a brass of similar composition coated with a dezincified surface around 1 µm-thick of apparently pure copper with a crust of cuprite and covellite on top. This indicates a differing corrosion environment for this link compared with the others – perhaps direct exposure to sea water rather than immersion in silt.

Overall, the measurements confirm the effectiveness over three decades of the different storage arrangements and conservation treatments applied to these artefacts – from the preventative removal of chlorides by soaking in distilled water through soaking in BTA to the interventive removal of concretion and corrosion followed by coating with BTA and silicone oil. This knowledge can inform the conservation strategies employed when treating such materials from a marine environment.

## Supplementary Material

Supporting Figures S1 to S7 and derivation of equation (1). DOI: 10.1107/S1600577520001812/ok5009sup1.pdf

## Figures and Tables

**Table 1:** Sample details The final column describes the sample at the time of analysis.

Sample number	Treatment on recovery	Appearance
MR81A1436	Ultrasonic cleaning in 10% *w*/*v* citric acid (Modalene) to dissolve any concretion. Scrubbing and use of a scalpel to remove largest areas of black corrosion/concretion. Further ultrasonic cleaning in Modalene and air abrasion of the twist. Soaking in BTA and coating with silicone oil	Dull brass. No obvious attack by *e.g.* bronze disease [Fig. 1 ▸(*a*)]
MR81A2249	Soaking in BTA aqueous solution [strength and time not recorded but likely in the range 1–5% and 2 weeks–1 day depending on the strength (Jones, 2003 ▸)].	Black with small blue–green patches. Red–brown where black layer had flaked off [Fig. 1 ▸(*b*)]
MR82A6000	Soaked in distilled water in 1989 with several changes (Jones, 2003 ▸), possibly at a temperature 50–80°C.	Dull brownish red friable surface, typical of cuprite (Cu_2_O) [Fig. 1 ▸(*c*)]

**Table 2:** Approximate mean atomic composition in wt% from SEM-EDS and XRF ‘–’ denotes undetected. The + and − values show the extremes.

	MR81A2249		MR81A1436		MR82A6000		
C	24	^+7^_−4_	22	^+2^_−2_	4.35	^+0.15^_−0.15_	
N	13	^+1.5^_−3.5_	Trace		4.2	^+0.4^_−0.4_	
O	20	^+4.5^_−5.4_	13	^+2^_−2_	8.4	^+1.4^_−1.4_	
Na	Trace†		Trace		–		
Mg	0.4	^+0.07^_−0.16_	–		–		
Al	0.5	^+0.2^_−0.2_	0.7	^+0.4^_−0.3_	–		
Si	0.7	^+0.3^_−0.4_	2	^+1.6^_−1.1_	0.28	^+0.09^_−0.09_	
P	Trace		–		–		
S	2.1	^+0.6^_−0.4_	0.46	^+0.1^_−0.1_	1.34	^+0.16^_−0.16_	
Cl	1.4†	^+1.7^_−1.1_	0.25	^+0.04^_−0.03_	–		
K	0.2	^+0.05^_−0.06_	0.14	^+0.02^_−0.03_	–		
Ca	0.8	^+0.19^_−0.24_	0.4	^+0.11^_−0.13_	0.24	^+0.08^_−0.08_	
Fe	1.2	^+0.74^_−0.78_	0.7	^+0.04^_−0.2_	1.12	^+0.41^_−0.41_	
Ni	Trace		Trace		–		
Cu	22	^+11^_−14_	45	^+4^_−5_	66.6	^+0.6^_−0.6_	
Zn	13	^+9^_−7.5_	16	^+1^_−2_	12.3	^+1.7^_−1.7_	
As	Trace		Trace‡		–		
Se	Trace		Trace‡		–		
Sn	–		–		1.1	^+0.01^_−0.01_	
Pb	Trace		Trace‡		–		

† Cl and Na correlated in one position.

‡ Levels on the conserved link are <0.1× those on the corroded link.

**Table:** 

	MR81A2249		MR81A1436		MR82A6000	
	*a* (nm)	Zn (wt%)	*a* (nm)	Zn (wt%)	*a* (nm)	Zn (wt%)
311	0.36761	28.170	0.36752	27.802	0.36718	26.402
222	0.36736	27.145	0.36730	26.898	0.36709	26.028

**Table:** 

	MR81A1436 + MR81A2249	MR82A6000
Average (wt%)	27.50 ± 1	26.2 ± 1
