# Supplementary material for: Synchrotron X-ray diffraction investigation of the surface condition of artefacts from King Henry VIII’s warship the Mary Rose
Source: J Synchrotron Radiat. 2020 Apr 15;27(Pt 3):653–63. doi: 10.1107/S1600577520001812 (PMC7206548; doi:10.1107/S1600577520001812)
Supplement: Supplementary file 1 [file s-27-00653-sup1.pdf]

JOURNAL OF SYNCHROTRON RADIATION

Volume 27 (2020)

Supporting information for article:

Synchrotron XRD investigation of the surface condition of artefacts from King Henry VIII's warship The Mary Rose

Mark G Dowsett, Pieter-Jan Sabbe, Jorge Alves Anjos, Eleanor J. Schofield, David Walker, Pam Thomas, Steven York, Simon Brown, Didier Wermeille and Mieke Adriaens

EXPERIMENTAL SECTION

Figure S1a shows the vice manufactured from acetal copolymer by the first author for this series of experiments. No metal comes into contact with the artefacts and the combination of prismatic jaws and surface pegs is adaptable to a large variety of shapes. The conserved link MR81A1436 can be seen resting securely around a peg prior to analysis. To the upper right of the image the Kapton® window on the ion chamber used as beam monitor can be seen.

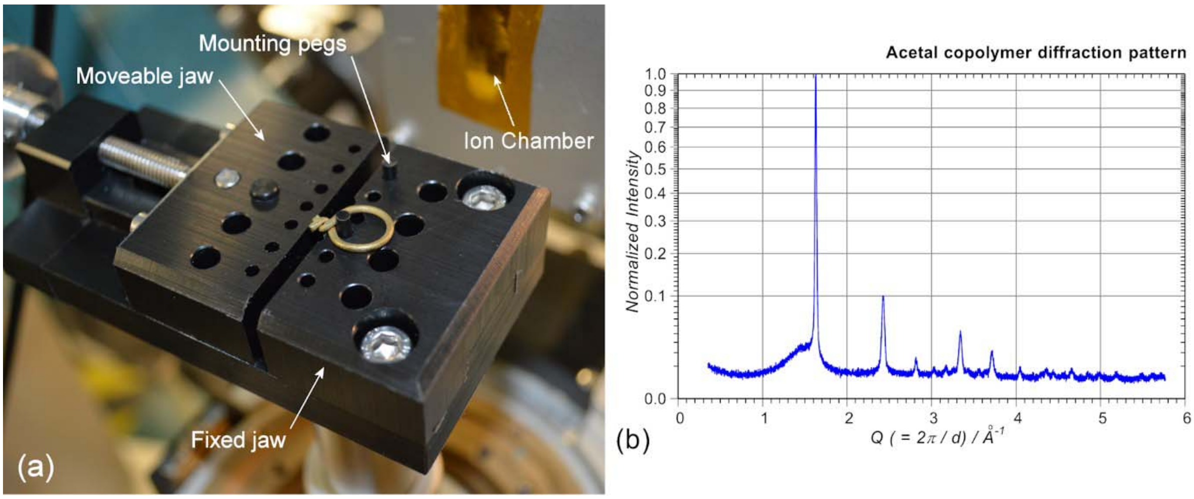

Figure S1: (a) The acetal copolymer vice. (b) X-ray diffraction pattern of the acetal copolymer (square root intensity scale).

Figure S1b shows the diffraction pattern of the material measured on the Panalytical Xpert Pro MPD at The University of Warwick for the purpose of eliminating any irrelevant peaks from the SR-XRD measurements. The most intense reflection was found on the SR-XRD

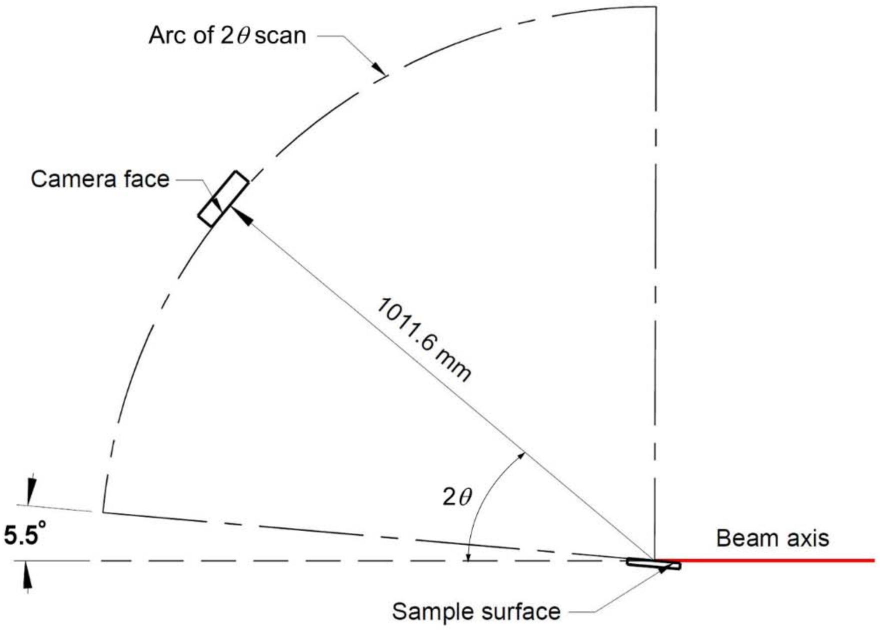

pattern for MR82A6000, the "copper" link and the first two reflections were detected in the SR-XRD for conserved link.

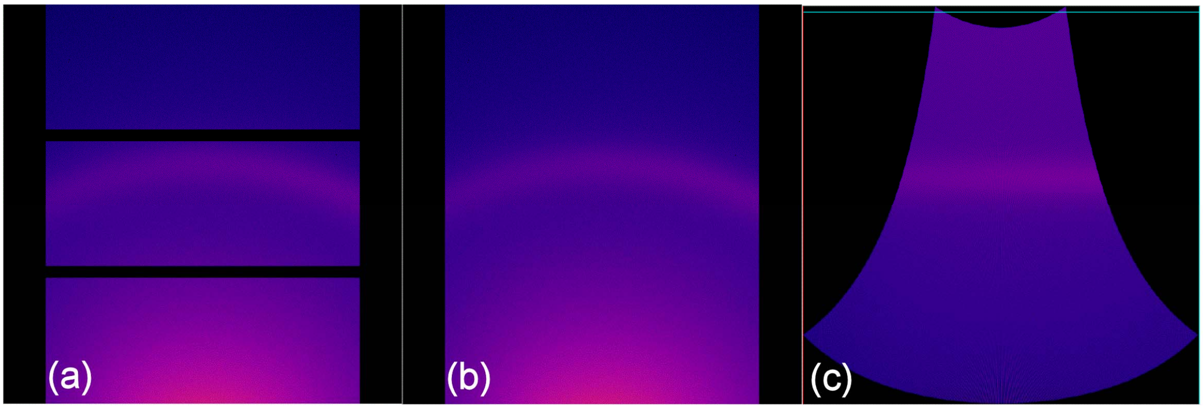

Figure S2: Geometry of SR-XRD measurements

Figure S2 shows the geometry of the SR-XRD measurements with the sample at 5.5° to the beam. The camera was moved in 81 steps around the arc shown to acquire a set of 82 images typically exposed for 100 s each.

Figure S3: (a) Image acquired with the camera at its starting angle of 5° (ironbow colour scale). Note the arc due to scattering from the Kapton beam monitor window up-beam of the

sample. (b) After gap filling operation. (c) The 2θ - γ map to be integrated into the final image.

Figure S3 summarizes the acquisition, gap filling and mapping operations of esaProject. The bright arc at 2θ= 5.6° has the correct radius for a Scherrer cone because the mapping shows it to be at constant 2θ. However it was observed on all the samples, irrespective of the material and is due to 002 scattering from the Kapton exit window on the beam monitor around 60 mm farther from the camera than the sample (true scattering angle at 8.5 keV ~5.3°).

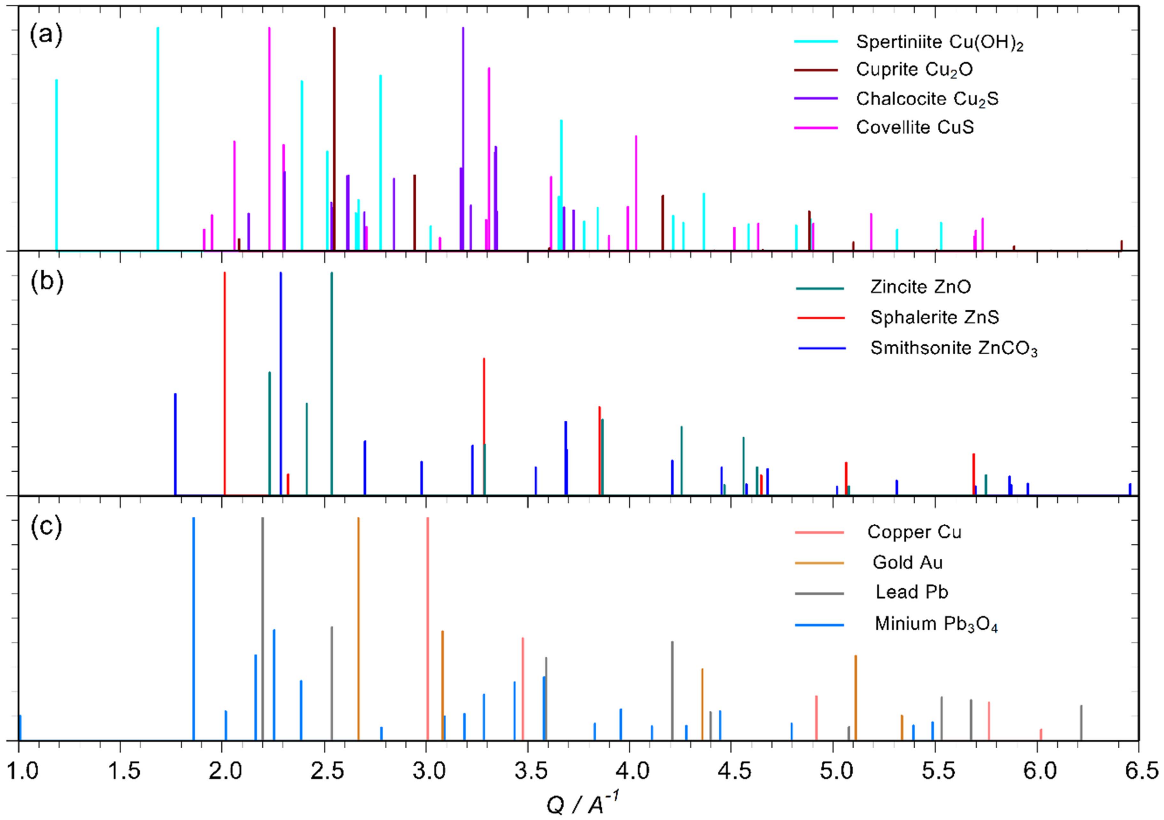

Figure S4: Stick patterns for the compounds identified on the surfaces of the Mary Rose samples. (a) copper compounds, (b) zinc compounds and (c) other metals and compounds.

RESULTS AND DISCUSSION

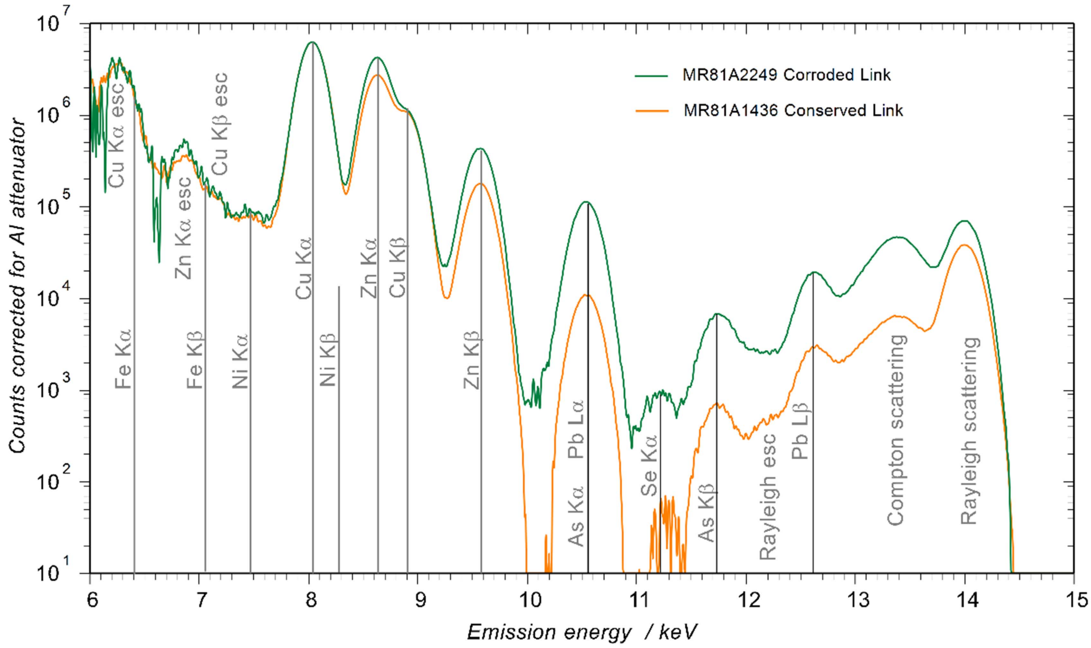

Figure S5: XRF data for the conserved and corroded links corrected for the energy dependent attenuation of 0.4 mm of Al foil and background subtracted. There is no useful signal below 6 keV because the transmission of the foil effectively falls to zero.

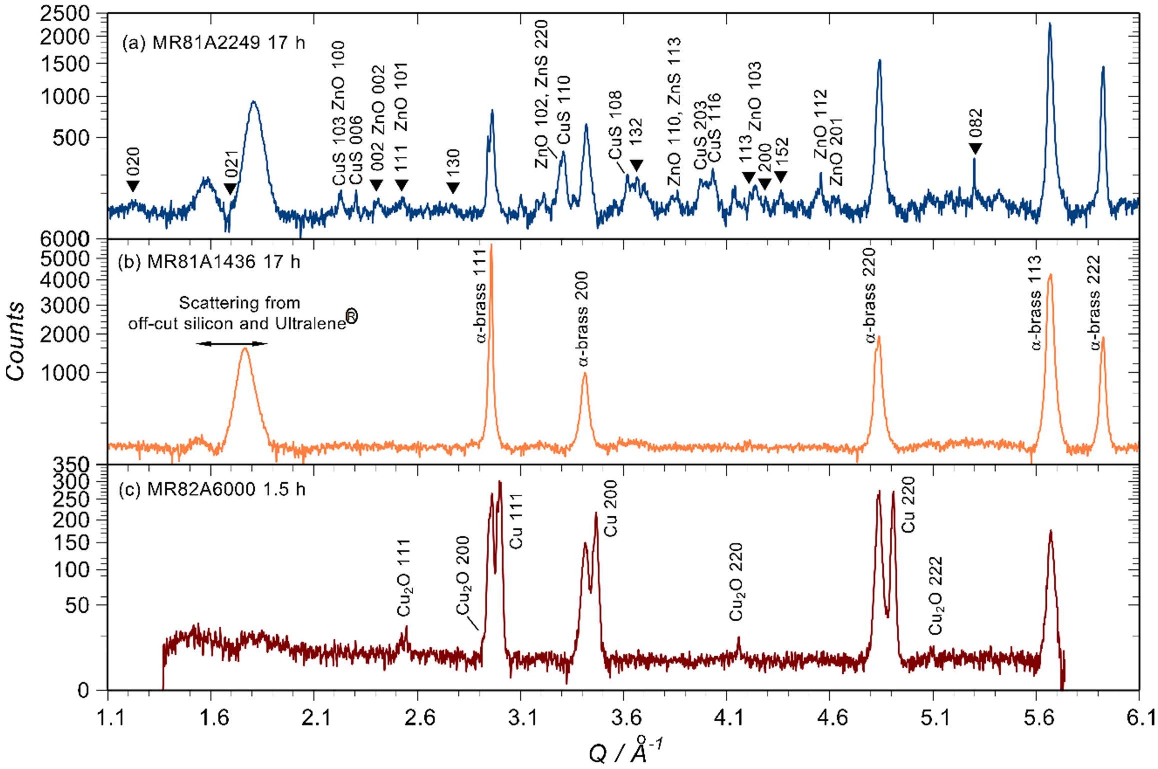

Figure S6: Laboratory XRD patterns obtained from the chain mail links. Spertiniite on the corroded link is indicated as ▼. Differences in relative peak intensities between these data and the SR-XRD are due to differences in beam energy, scan geometry, incident angle, changes in flux from the variable aperture used in the laboratory work, and the fact that the above data represent an average over the whole of the upper side of the sample.

Figure S7: Parabolic fit to data from Rendle (1981) and Voncken & Verkroost (1997) for Zn wt% as a function of lattice constant. A parabolic rather than linear fit has been used because Vegard's law rarely applies to metallic solid solutions (Suryanarayana & Norton, 1998). Rendle used a similar fit although the curve is not specified.

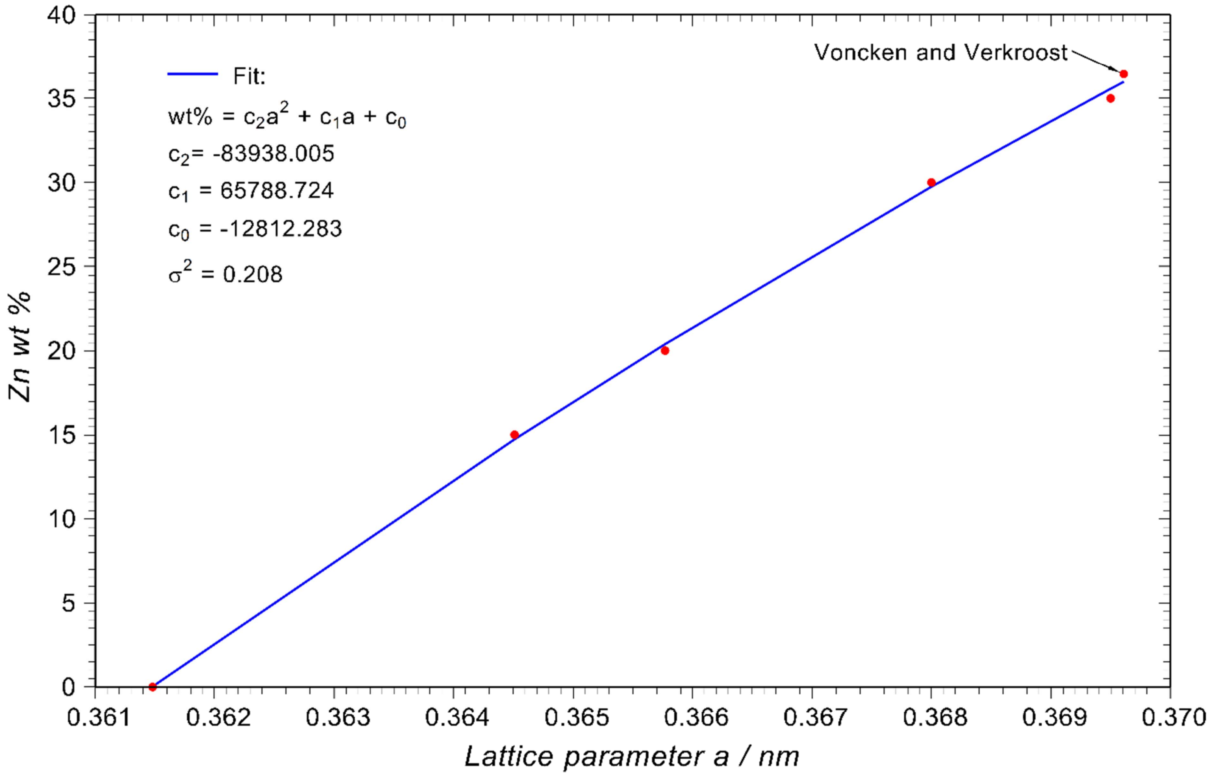

APPENDIX I

Derivation of Equation 1

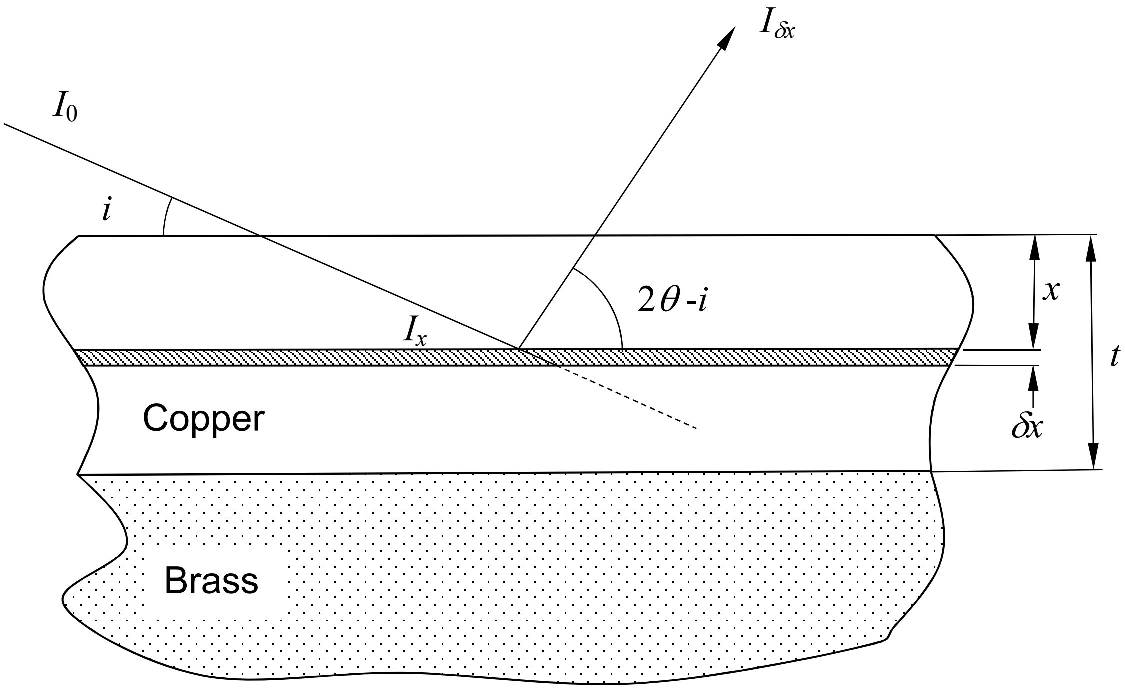

Consider a layer of copper with average thickness t on a brass substrate: X-rays intensity I_0 are incident at an angle i to the surface. The x-ray intensity I_x arriving at a layer thickness δx at a depth x below the surface is given by

$$I_x = I_0 \exp\left(-\frac{x}{\Lambda \sin i}\right),$$

where Λ is the x-ray attenuation length.

The intensity scattered out of the thin layer at a scattering angle 2θ will be:

$$I_{\delta x} = K_{Cu} I_x \delta x \exp\left(-\frac{x}{\Lambda \sin(2\theta - i)}\right),$$

where K_{Cu} is the scattering constant for copper. Then

$$I_{\delta x} = K_{Cu} I_0 \delta x \exp\left\{-\frac{x}{\Lambda}\left[\frac{1}{\sin i} + \frac{1}{\sin(2\theta - i)}\right]\right\}.$$

The scattered intensity I_t from a layer of thickness t amounts to

$$I_t = K_{Cu} I_0 \int_0^t \exp\left\{-\frac{x}{\Lambda}\left[\frac{1}{\sin i} + \frac{1}{\sin(2\theta - i)}\right]\right\} \mathrm{d}x$$
$$= \frac{-K_{Cu} I_0 \Lambda}{\frac{1}{\sin i} + \frac{1}{\sin(2\theta - i)}} \left(\exp\left\{-\frac{t}{\Lambda}\left[\frac{1}{\sin i} + \frac{1}{\sin(2\theta - i)}\right]\right\} - 1\right).$$

For solid copper, $t \to \infty$ so that

$$I_\infty = \frac{K_{Cu} I_0 \Lambda}{\frac{1}{\sin i} + \frac{1}{\sin(2\theta - i)}}$$

Taking the ratio I_t / I_∞ we get

$$\frac{I_t}{I_\infty} = 1 - \exp\left\{-\frac{t}{\Lambda}\left[\frac{1}{\sin i} + \frac{1}{\sin(2\theta - i)}\right]\right\}$$

so that finally

$$t = -\frac{\Lambda}{\frac{1}{\sin i} + \frac{1}{\sin(2\theta - i)}} \ln(1 - R) \text{ where } R = \frac{I_t}{I_0}.$$
